# Psychiatric evaluation of children and adolescents’ victims of the ISIS

**DOI:** 10.1192/j.eurpsy.2023.1546

**Published:** 2023-07-19

**Authors:** N. Uzun, H. Ferahkaya, Ö. F. Akça, A. Bilgiç

**Affiliations:** 1 Child and Adolescent Psychiatry, Necmettin Erbakan University Meram School of Medicine; 2 Child and Adolescent Psychiatry, Dr.Ali Kemal Belviranlı Maternity and Childrens’ Hospital, Konya; 3 Child and Adolescent Psychiatry, Private Practice, İzmir, Türkiye

## Abstract

**Introduction:**

Psychiatric symptoms of children and adolescents’ people whose families have joined the Islamic State of Iraq and Syria (ISIS) for various reasons, lived under the threat of terrorism in or outside Turkey, lost their parents or were separated for various reasons, are not clearly known.

**Objectives:**

This research aims to psychiatrically evaluate children and adolescents who are victims of various reasons due to their families joining the ISIS.

**Methods:**

31 children and adolescents living with or without parents in Turkey, whose parents had joined ISIS, were included in the study. The sociodemographic data of the participants, the country they were in during their time in ISIS, the current situation of their parents, their exposure to terror attacks, their stay in prison were determined. Psychiatric diagnoses of the participants were determined according to DSM-5. Afterwards, the participants and caregivers were asked to fill out the Revised Child Anxiety and Depression Scale Child and Parent Form (RCADS-C, RCADS-P), Child Post Traumatic Stress Disorder Reaction Index Scale (CPTS-RI), Child and Youth Resilience Measure (CYRM) and Conners Parental Rating Scale Revised Short Form (CPRS-SF)

**Results:**

14 (45.2%) of the participants were boy and 17 (54.8%) were girl. The mean age of the participants was 10.55±3.79. The average age of the participants’ families at the time they joined ISIS was 4.81±3.37 years. The average age of the participants to return to our country was 8.87±3.99. All of the participants remained in Iraq. 25 (80.6%) of the participants stayed at home and 6 (19.4%) of them stayed in the camp. 19 (61.3%) of the participants were exposed to at least one conflict, 12 (38.7%) witnessed at least one terror attack, 5 (16.1%) were injured, 26 (83.9%) were not injured. It was found that 28 (90.3%) of the participants remained in prison and 3 (9.7%) did not. Participants’ RCADS-C scores were 23.42±15.96, RCADS-P total scores were 16.25±16.12, CPTS-RI scores 20.3±14.56. CYRM total scores were 46.65±9.04, and CPRS-SF total scores were 19.74±16.55.

**Conclusions:**

It has been revealed that there are various psychiatric problems of varying severity in children and adolescents who are victims of ISIS in Turkey.

**Disclosure of Interest:**

None Declared

